# Unambiguous Interpretation of the Pathogenicity of the GLA c.547+3A>G Variant Causing Fabry Disease

**DOI:** 10.3390/genes15091212

**Published:** 2024-09-17

**Authors:** Mario Urtis, Claudia Cavaliere, Viviana Vilardo, Chiara Paganini, Alexandra Smirnova, Carmelina Giorgianni, Alessandro Di Toro, Luisa Chiapparini, Carlo Pellegrini, Maurizia Grasso, Eloisa Arbustini

**Affiliations:** 1 Centre for Inherited Diseases, Department of Research, Fondazione IRCCS Policlinico San Matteo, 27100 Pavia, Italy; m.urtis@smatteo.pv.it (M.U.); cl.cavaliere@smatteo.pv.it (C.C.); vi.vilardo@smatteo.pv.it (V.V.); c.paganini@smatteo.pv.it (C.P.); a.smirnova@smatteo.pv.it (A.S.); c.giorgianni@smatteo.pv.it (C.G.); a.ditoro@smatteo.pv.it (A.D.T.); m.grasso@smatteo.pv.it (M.G.); 2 Neuroradiology Unit, Fondazione IRCCS Policlinico San Matteo, 27100 Pavia, Italy; l.chiapparini@smatteo.pv.it; 3 Clinical-Surgical, Diagnostic and Pediatric Sciences Department, University of Pavia, 27100 Pavia, Italy; 4 Division of Cardiac Surgery, Cardiotoracovascular Department, Fondazione IRCCS Policlinico San Matteo, 27100 Pavia, Italy; c.pellegrini@smatteo.pv.it

**Keywords:** Anderson–Fabry disease, endomyocardial biopsy, electron microscopy, immunohistochemistry, variant interpretation

## Abstract

Objectives: This study aims to demonstrate the role of case-level American College of Medical Genetics (ACMG) criteria, such as familial segregation and pathology data, in providing conclusive evidence for the pathogenicity of ultrarare *GLA* variants causing Anderson–Fabry disease when gene-level and variant-level criteria provide ambiguous or discrepant results. Case/family description: A 52-year-old woman presented with new-onset shortness of breath, chest pain, and palpitations. Echocardiography revealed mild left ventricular wall thickening (14 mm) and mild diastolic dysfunction. She was the second of three siblings born to unrelated parents, both of whom died from malignancies. Family screening identified brothers, one affected 55-year-old with hypertension and asthma and one unaffected 47-year-old. The 15-year-old son of the proband complained of exercise-induced burning feet acral pain his electrocardiogram showed a short PR interval and signs of early hypertrophy. Results: Endomyocardial biopsies of the proband and the affected sibling demonstrated substrate accumulation (globotriaosylceramide). The anti-α-galactosidase-A immunostain showed a total loss of the enzyme in the hemizygous male and a mosaic pattern in the heterozygous female. The next-generation sequencing short-read multigene panel identified the c.547+3A>G variant in the *GLA* gene and excluded variants in other genes; Oxford-Nanopore long-read sequencing excluded known pathogenic deep intronic variants. A Multiplex-Ligation-dependent-Probe-Amplification assay excluded copy number variations. Based on the variant-level and gene-level ACMG criteria, the variant was classified as a Variant of Uncertain Significance or Likely Benign using different bioinformatic tools. By adding case-level functional data (endomyocardial biopsy, PS3_VeryStrong) and familial data (segregation of genotype with phenotype, PP2_Moderate), the variant was classified as Likely Pathogenic/Pathogenic. Conclusion: ACMG case-level data can unambiguously resolve uncertain interpretations of *GLA* variants.

## 1. Introduction

Anderson–Fabry Disease (AFD) is a rare X-linked syndrome caused by pathogenic defects in the *GLA* gene encoding the enzyme α-galactosidase A (α-Gal A) (ICD-10: E75.2; ORPHA324; RCG080; MIM#301500) [1]. The resulting enzyme deficiency leads to intracellular accumulation of undigested globotriaosylceramide (GB3) with consequent organ/tissue damage. In classical AFD, the diagnosis can be suspected based on the multiorgan/system phenotypical traits and be supported by enzyme activity test in affected hemizygous males but not in heterozygous females in whom genetic testing is essential for the diagnosis because enzyme activity levels are not informative [2,3]. In non-classical, late-onset AFD, clinical manifestations may involve a single organ, typically the heart. Alternatively, the diagnosis of AFD can result from screening studies (α-Gal A enzyme activity or genetic testing) in patients with the following “at-risk” phenotypes [4,5,6]: cardiac (hypertrophic cardiomyopathy, HCM) [7], nervous, either central (cryptogenic stroke) or peripheral (peripheral neuropathy) [8], renal (albuminuria, proteinuria, renal failure) [9], dermatological (hypohidrosis, angiokeratomas) [10], ocular (non-iatrogenic cornea verticillata) [11,12], or gastroenterological [13,14]. In patients with late-onset cardiac variants (e.g., p.N215S, p.F113L), the heart is the main and nearly uniquely affected organ, mimicking HCM [4] and representing 0.94% males and 0.90% females in a consecutive series of patients clinically diagnosed with HCM [6].

The ACMG/AMP criteria-based interpretation of the pathogenicity of *GLA* variants [15] distinguishes clinically actionable Pathogenic/Likely Pathogenic (P/LP) variants from non-clinically actionable variants that include both Variants of Uncertain Significance (VUS) and Likely Benign/Benign variants (LB/B) [16]. The pathogenicity is calculated with a scale point system that results from the sum of fulfilled criteria weighted by their strength of evidence [17]. Variant interpretation software automatically analyzes variant-level and gene-level criteria (nine out of sixteen pathogenic and eight out of twelve benign criteria) based on gene features, variant types, impact prediction, prevalence in case series and control populations, and reputable literature, irrespective of clinical and genetic evaluations of the patients and families (a priori criteria). Vice versa, patient-level criteria require non-automated curation of phenotypical traits, clinical genetics, and functional data. When ultra-rare or novel variants (unreported in GnomAD, ClinVar, etc.) are identified, the use of different software can provide discrepant interpretations of the same variants, either over- or under-estimating the class of pathogenicity. The addition of patient-level criteria, in particular with Strong or Very Strong strength, may equalize different interpretations. The PS3 criterion (Pathogenic with Strong strength) may add conclusive functional evidence of the deleterious impact of the variants based on in-vitro experiments or in-vivo tissue studies, in the absence of a second variant in the same gene and in known genes that can mimic similar pathophysiology [16]. On the contrary, the BS3 (Benign with Strong weight) criterion excludes the damaging effect of a gene variant. In AFD, the in vivo PS3 criterion is achievable by demonstrating the presence of intracellular accumulation and its specific composition using targeted light microscopy immunohistochemistry and immune-electron microscopy techniques [6,14,16].

We describe an ultrarare variant in the *GLA* gene identified with next-generation sequencing (NGS) multigene panel testing in a female proband with a clinical diagnosis of suspected mild “HCM”: the interpretation of the pathogenicity based on gene- and variant-level criteria activated by different software remained ambiguous. The female sex prevented the diagnostic contribution of enzymatic activity. The immunohistochemical study of the endomyocardial biopsy (EMB) added the PS3 criterion with a Very Strong level of evidence proving the pathogenicity of the *GLA* variant and the conclusive diagnosis of Fabry Disease.

## 2. Materials and Methods

### 2.1. EMB and Right Heart Catheterization

Right ventricular EMB was performed according to the Stanford procedure, along with right heart catheterization in both proband and brother, as per institutional protocol. Six myocardial samples were obtained from each patient; four samples were processed for light microscopy and two samples for electron microscopy [6].

### 2.2. Light Microscopy Immunohistochemistry

For the detection of the substrate (GB3), the tissue sections were deparaffinized and rehydrated before pretreatment for epitope unmasking by microwave heating the slides in Tris/EDTA buffer (pH 9.0); then, the slides were incubated with anti-GB3 primary antibody (TCI Chemicals, Zwijndrecht, Belgium, product number A2506) diluted 1:1000, overnight at 4 °C in a humidified chamber. Slides were washed and incubated with DAKO EnVision+ System-HRP Labeled Polymer Anti-Mouse for 30 min. The signal was detected using an HRP substrate, and slides were counterstained with Harris hematoxylin and mounted using Bio Mount HM (Bio-Optica, Milan, Italy). The immunohistochemical detection of the α-Gal A enzyme was performed with the automated Leica Bond RX using Bond Polymer Refine DAB IHC Protocol using the pre-treatment 10’ ER1 (pH6), and incubation with anti-galactosidase α primary antibody (Atlas Antibodies, Stockholm, Sweden, product number APA000237) diluted 1:50. The signal was detected using the Bond Polymer Refine DAB Detection Revelation System. Images were acquired using the Leica ScanScope slide scanner (Leica Biosystems Italia, Buccinasco, Italy) and the Aperio Image Scope software (v. 9.1.0.1567).

### 2.3. Electron- and Immuno-Electron Microscopy Study

Ultrastructural studies of EMBs were performed per institutional diagnostic protocol. Samples were fixed with Karnovsky’s solution, post-fixed with cold 1% OsO4 in 0.1 mol/L cacodylate buffer, pH 7.3, dehydrated in ethanol and propylene oxide, and embedded in Epon–Araldite resin overnight at 60 °C. Ultra-thin sections (600- to 800-Å-thick) were mounted on nickel grids and stained with lead citrate, 5% uranyl acetate, and Reynold’s solution.

Post-embedding immuno-electron microscopy was performed according to standardized, validated methods [6,14]. Ultrathin sections were mounted on parlodion-coated nickel grids. Sections were incubated overnight (4 °C) with the primary anti-GB3 (in mouse) antibody and then incubated for 1 h with the secondary antibody (anti-mouse IgG) conjugated to 15 nm colloidal gold particles as a revealing system (British Biocell International, Cardiff, UK). Sections were stained with uranyl acetate 5% and Reynold’s solution. The specificity of immunoreactions was controlled using normal serum as a primary antibody. Sections were examined with a JEOL JEM-1011 electron microscope (JEOL ITALIA S.P.A, Milan, Italy).

### 2.4. Genetic Test, NGS Sequencing and Data Analysis

The genetic test was carried out on DNA extracted from peripheral blood samples. We used both short-read Illumina technology and Oxford Nanopore Technology (ONT) for the identification of single nucleotide variants (SNVs), insertions/deletions (InDels), and copy number variants (CNVs), while a Multiplex Ligation-dependent Probe Amplification (MLPA) assay was used for the identification of CNVs. ONT sequencing was carried out to expand the target regions beyond exons and flanking regions, to assess the presence of the deep intronic variants (Table 1) that could be missed by short-read sequencing technology.

The libraries for Illumina sequencing were prepared using an Agilent SureSelect HS2 custom panel (Agilent Technologies, Santa Clara, CA, USA) of 124 genes associated with genetic cardiomyopathies. The sequencing was performed on the Illumina NextSeq1000 platform (Illumina, San Diego, CA, USA) using a P1 sequencing kit. The data were analyzed using an institutionally validated in-house pipeline.

The libraries for ONT sequencing were prepared using the CE-IVD CardioPRO kit provided by 4bases “https://4bases.ch (accessed on 13 August 2024)”, which uses ONT V14 chemistry. Long-read sequencing was run using an R10.4.1 flowcell in the ONT GridION platform. The base calling was performed using the Dorado Super accurate basecaller model. Alignment and variant calling (SNVs, short InDels, and CNVs) were performed via the 4eVAR pipeline for ONT data “https://4evar.4bases.ch (accessed on 13 August 2024)”. The 4eVAR pipeline (v. 2.0) is a bioinformatic analysis software that guarantees maximum performance with 4bases diagnostic kits.

*GLA* variants were confirmed by Sanger sequencing as a further validation analytical tool, as per local guidelines, using the 3500 Genetic Analyzers Dx sequencer (Thermo Fisher Scientific, Waltham, Massachusetts, USA). Finally, MLPA analysis was performed on 3500 Genetic Analyzers Dx using the SALSA MLPA Probemix P159 *GLA* kit (MRC Holland, Amsterdam, Holland) to further exclude the presence of CNVs in the *GLA* gene.

### 2.5. Variant Interpretation

Variants identified by both Illumina and ONT technology were interpreted using Franklin v.2022.6 “https://franklin.genoox.com/ (accessed on 13 August 2024)” and Varsome v.11.10.0 “https://varsome.com/ (accessed on 13 August 2024) software and our interpretation workflow (OSM), which adopts the scale point system for the pathogenicity classification [17].

Both Franklin and Varsome implement an ACMG pathogenicity classification framework with a semi-automated evaluation of variant-level and gene-level criteria, while case-level criteria were curated after morphofunctional tissue studies and clinical and genetic family screening. Our workflow considered the PP3 criterion to be met with supporting strength if both MaxEntScan [18] and SpliceAI [19] tools concordantly predicted the variant to cause aberrant splicing.

Case-level criteria were curated as follows: the PP1 criterion was met in the presence of at least three family members supporting the segregation of the *GLA* c.547+3A>G variant and the disease (affected and variant carrier or healthy and non-carrier), and the strength was upgraded to moderate with at least five family members supporting the segregation. The strength of PS3 was upgraded to Very Strong when electron and light immune-microscopy proved the specific GB3 composition of the intra-myocyte stored material, demonstrating the *GLA* defect-related cause of the disease and the expression of α-GAL A in the immune assay confirmed the absence of protein in the affected hemizygous male and the mosaic-like pattern in the female proband.

## 3. Results

### 3.1. Clinical History and Phenotype in the Proband

**Figure 1:**
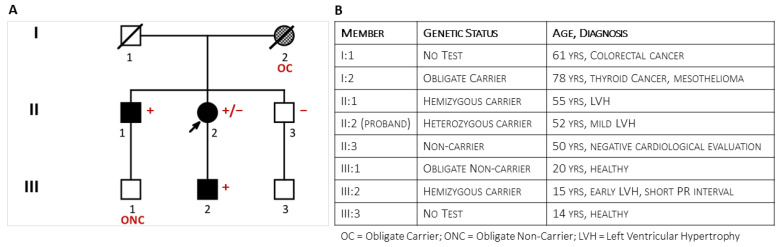
The figure shows the pedigree (Panel (**A**)) of the family at the end of the clinical and genetic family screening and specifies the family members’ phenotypes (Panel (**B**)).

A 52-year-old woman (Figure 1, II:2) was referred to the emergency room for a de novo episode of shortness of breath, atypical chest pain, and palpitations. Her cardiovascular history was silent, but the baseline electrocardiogram (ECG) showed diffuse negative T waves. The traditional risk profile did not show significant comorbidities or risk factors except untreated borderline hypertension. Coronary Computed Tomography Angography (CCTA) demonstrated patent coronary arteries. The two-dimensional transthoracic echocardiography (2DTTE) showed slightly increased, symmetrical left ventricle wall thickness (14 mm) and mild diastolic dysfunction in the absence of regional wall motion abnormalities. Deep clinical phenotyping did not add specific contributors to the diagnostic hypothesis.

### 3.2. Clinical Family Screening

The proband was the second of three sibs from unrelated parents. Her father (I:1, Figure 1) had died at 61 years from colorectal cancer. The mother (I:2) survived thyroid cancer and died at the age of 78 from mesothelioma; she was reported to have been hypertensive. The 55-year-old brother had a history of hypertension and allergic asthma. As a blood donor, he was undergoing regular clinical monitoring. Shortly after the onset of symptoms in the sister, he was incidentally diagnosed with symmetrical LV hypertrophy (15 mm) and mild mitral valve regurgitation. His stress test was negative. Later echocardiographic evaluation confirmed left ventricular (LV) hypertrophy (2DTTE: Interventricular Septum thickness = 16 mm; LV Posterior wall = 18 mm). Cardiac magnetic resonance showed an LV thickness ranging from 17 mm to 20 mm at the apical level and extensive non-ischemic late gadolinium enhancement. Coronary arteries were angiographically patent. He was prescribed ACE-inhibitors, beta blockers, Apixaban, and maintenance with Fluticasone furoate/vilanterol for asthma. Brain Magnetic Resonance (BMR) imaging did not show abnormalities. Deep clinical phenotyping did not add specific contributors to the diagnostic hypothesis: liver, kidney, and thyroid functions were normal; dermatologic evaluation showed scattered ruby angiomas; and ophthalmologic evaluation (fundus, cornea, and retina) confirmed known hypermetropia without other significant findings. The clinical and instrumental evaluation of the asymptomatic youngest brother (47-year-old) (II:3) did not show cardiac and extracardiac abnormalities. Clinical evaluation of family members III:1 and III:3 was negative. Vice versa, the 15-year-old son of the proband (III:2) described exercise-induced burning arthralgia of the feet; his electrocardiogram showed a short PR interval (94 msec), a Sokolow–Lyon index of 39 mm (suggesting early hypertrophy), isolated premature ventricular contractions, and diphasic T waves in anterior leads.

### 3.3. Genetic Test in the Proband and Relatives

Short-read Illumina NGS of a multigene panel in the proband identified the c.547+3A>G ultrarare non-canonic splice variant in the *GLA* gene. The MLPA test confirmed the absence of CNVs in *GLA*. The proband’s son (III:2) and her older brother (II:1) tested positive for the variant, while the youngest brother (II:3) tested negative. A second analytical confirmation with long-read Oxford Nanopore sequencing performed both on the proband and her affected brother (II:1) excluded known pathogenic deep intronic variants (e.g., the c.640−801G>A and the c.640−859C>T variants) in the *GLA* gene [20]. The deceased mother (I:2) was the obligate carrier who transmitted the *GLA* variant to the daughter (II:2) and son (II:1). The young son (III:1) of the firstborn brother (II:1) was an obligate non-carrier.

### 3.4. Endomyocardial Biopsy

**Figure 2:**
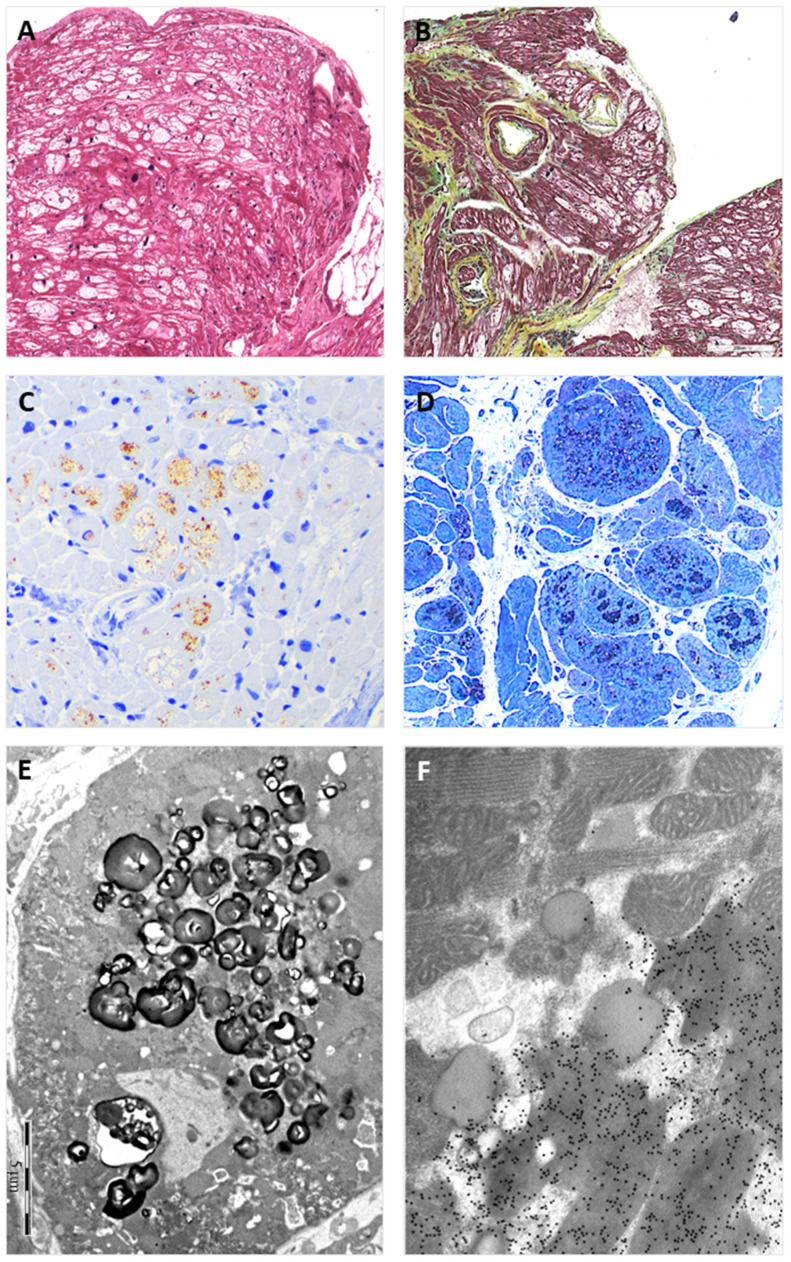
The figure shows EMB findings from the heterozygous proband (II:2). Panel (**A**) shows the typical view of the EMB with optically empty myocytes (H&E stain); Panel (**B**) shows common accompanying, non-substitutive interstitial fibrosis (Movat stain); Panel (**C**) shows intra-myocyte storage material specifically immuno-stained with anti-GB3 antibodies; Panel (**D**) shows a toluidine-blue stained semi-thin resin section with the intra-sarcoplasmic substrate accumulation (dark blue); Panel (**E**) shows the typical osmiophilic lamellar bodies substrate accumulation that are specifically immunogold-labeled with anti-GB3 antibodies in Panel (**F**).

**Figure 3:**
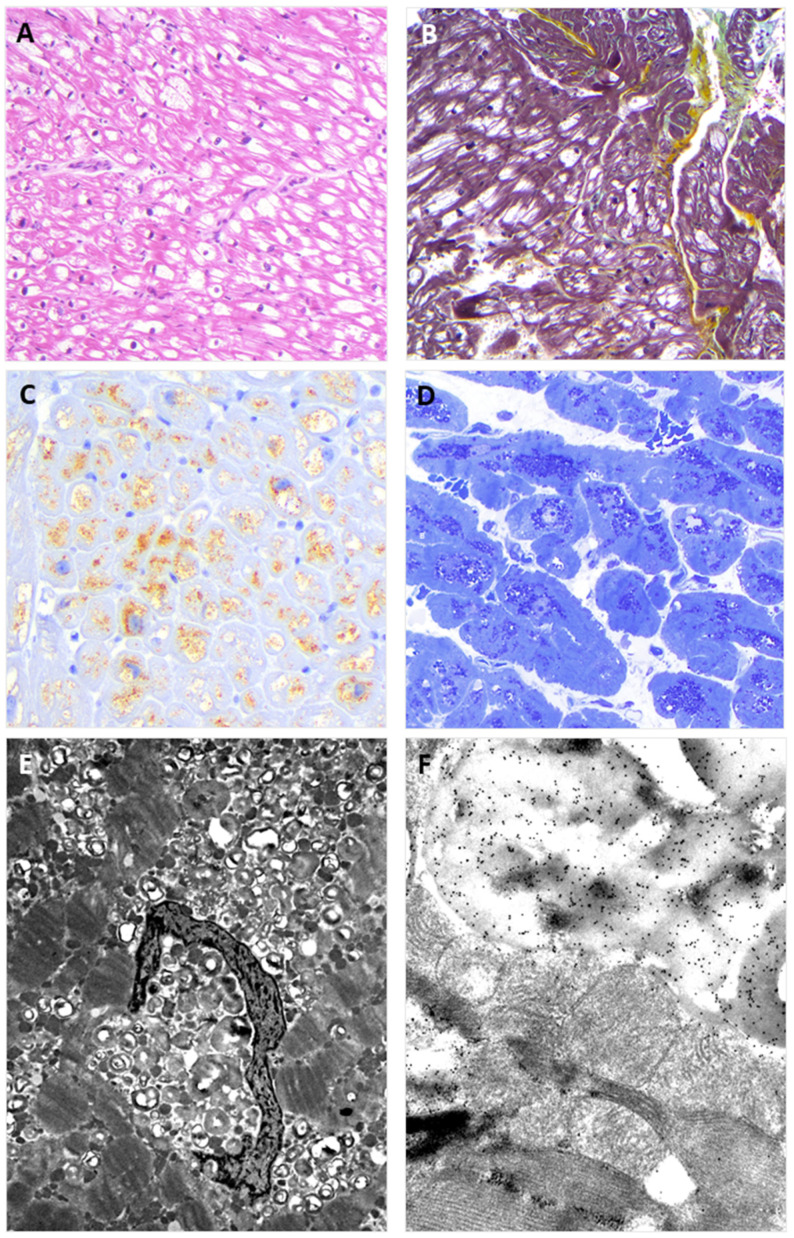
The figure shows EMB findings from the hemizygous proband brother (II:1). The findings replicate those observed in the EMB of the sister. Panel (**A**) shows the typical view of the EMB with optically empty myocytes (H&E stain); Panel (**B**) shows interstitial fibrosis commonly observed in the affected myocardium (green) (Movat stain); Panel (**C**) shows intra-myocyte storage material specifically immune-stained with anti-GB3 antibodies; Panel (**D**) shows toluidine-blue stained semi-thin resin section with the intra-sarcoplasmic substrate accumulation (dark blue); Panel (**E**) shows the typical osmiophilic lamellar substrate accumulation that is specifically immunolabeled with anti-GB3 antibodies in Panel (**F**).

**Figure 4:**
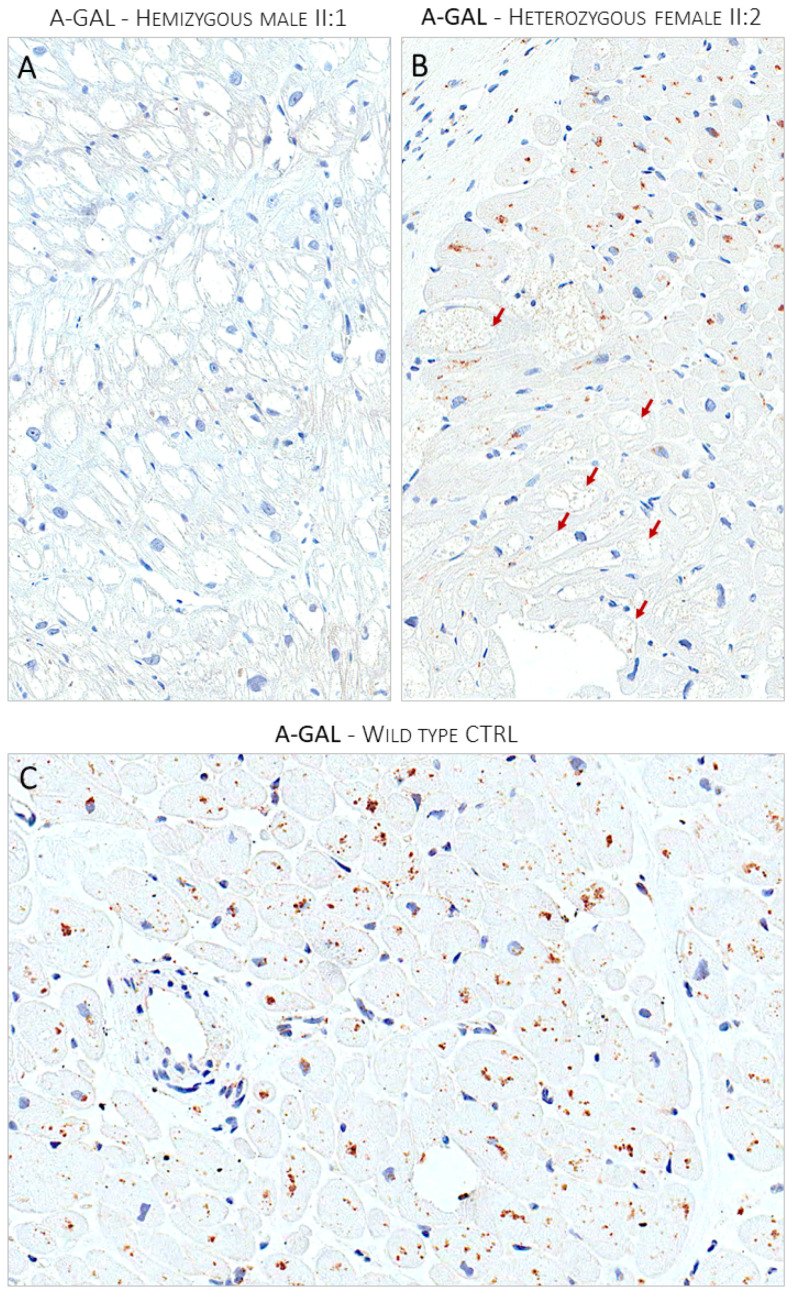
Panel (**A**) shows the lack of expression of α-Gal A enzyme immunostain in optically empty myocytes of the hemizygous affected male (II:1). Panel (**B**) shows the mosaic pattern of expression of α-Gal A enzyme in the heterozygous affected female proband (II:2). The enzyme expression is nearly absent in optically empty myocytes indicated by red arrows. Panel (**C**) shows the ubiquitous expression of the a-Gal enzyme in a control myocardial sample from a donor heart.

The proband agreed to undergo right heart catheterization and EMB, which was investigated with both light and electron microscopy, according to institutionally validated diagnostic protocols [6,14]. The routine light microscopy (LM) stain with hematoxylin–eosin (H&E) showed the pattern of “optically empty” myocytes that characterizes intra-sarcoplasmic storage diseases. The light microscopy immunostain with anti-GB3 antibodies specifically labeled the accumulated intra-myocyte material. The ultrastructural study demonstrated the typical dense osmiophilic lamellar bodies that constitute the pathological signature of the disease. The post-embedding immuno-electron microscopy study showed the accumulated substrate specifically labeled by anti-GB3 antibodies (Figure 2). The EMB performed in the brother (II:2) carrier of the sister variant demonstrated the same findings (Figure 3). The LM immuno-stain with anti-α-Gal A antibodies tested negative in the hemizygous male and showed a mosaic immunolabeling pattern in the heterozygous female (Figure 4).

### 3.5. Variant Classification

Variant-level and gene-level data:

The *GLA* c.547+3A>G variant is absent in gnomAD and in the LVOD *GLA* database “https://databases.lovd.nl/shared/transcripts/00008556 (accessed on 13 August 2024)” and is reported as VUS in ClinVar with a single submission (id: RCV001368906). The interpretation software provides different variant-level and gene-level–based interpretations. Using two of the most common software and our institutionally validated interpretation pipeline (OSM), the c.547+3A>G variant is classified as:

- VUS, when using Franklin (v.2022.6) software that activates the PM2 criterion with moderate strength (extremely low frequency in gnomAD population databases), and PP3 (for a missense or a splicing region variant, computational prediction tools unanimously support a deleterious effect on the gene, based on the latest recommendations for PP3/BP4 rules) with supporting strength “https://franklin.genoox.com/clinical-db/variant/snp/chrX-100656617-T-C (accessed on 13 August 2024)”;
- Likely Benign, when using Varsome (v.11.10.0), which activates the PM2 criterion with supporting strength and the BP4 criterion with moderate strength (multiple lines of computational evidence suggest no impact of gene or gene product) “https://varsome.com/variant/hg19/chrX-100656617-T-C (accessed on 13 August 2024)”.
- VUS, when using OSM, which activates the PM2 criterion with moderate strength, and the PP3 criterion (MaxEntScan splice effect variation of −75.82% and SpliceAI Donor Loss of 0.81; High impact).

**Figure 5:**
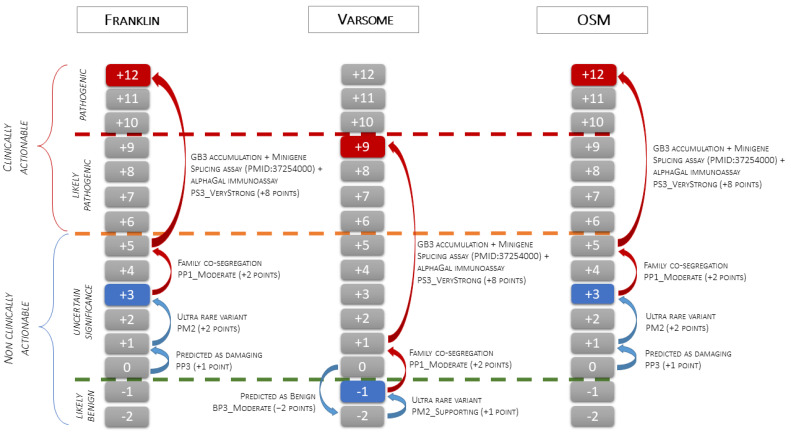
The figure illustrates the application of the scale point system of the ACMG classification to the c.547+3A>G *GLA* variant. The green dashed line indicates the threshold between the Likely Benign and VUS classes, the orange dashed line indicates the threshold between the VUS and Likely Pathogenic classes, and the red dashed line indicates the threshold between the Likely Pathogenic and Pathogenic classes. The blue boxes indicate the pathogenicity class obtained using only variant-level and gene-level data (blue arrows). Starting from 0 points, Franklin and OSM activate PP3 (+1 point) and PM2 (+2 points) and classify the variant as VUS (3 points), while Varsome activates BP3_Moderate (−2 points) and PM2_Supporting (+1 point) and classifies the variant as Likely Benign (−1 point). The red boxes indicate the final classification obtained after the addition of criteria based on case-level data (red arrows). Family co-segregation meets the PP1_Moderate criterion (+2 point), and EMB functional data, confirmed by splicing assay from PMID: 37254000, meet the PS3_VeryStrong criterion (+8 points). Adding case-level criteria, the final classification changes from VUS to Pathogenic using Franklin and OSM (3 + 10 = 13 points capped to 12 points) and from Likely Benign to Likely Pathogenic using Varsome (−1+ 10 = 9 points).

Case-level data: The segregation study met the PP2 criterion with moderate strength (three affected variant carriers and three healthy *GLA* negative family members, see the Methods section). The functional data met the PS3 criterion with Very Strong strength. The presence of myocardial GB3 accumulation specifically labeled with anti-GB3 antibodies, the pattern of the α-Gal A immunostaining in the proband, and the absence of the enzyme expression in the brother, proved the damaging effect of the variant that causes the loss of function of the *GLA* gene due to haplo-insufficiency. The addition of the criteria based on case-level data shifted the class from VUS (Franklin and OSM) or LB/B (Varsome) to LP/P (Figure 5).

## 4. Discussion

Our cases demonstrate how tissue studies, in particular, myocardial tissue in the case of cardiac clinical manifestations, provide unambiguous evidence and precise diagnosis of AFD cardiomyopathy when the genetic test identifies ultrarare or novel gene variants classified as VUS using a priori ACMG pathogenicity criteria. Our proband is a 52-year-old woman without clinical indications for eventually performing an extracardiac biopsy who is a heterozygous carrier of a non-otherwise classifiable VUS in the *GLA* gene. Her EMB findings validated the pathogenicity and disease mechanism of the c.547+3A>G *GLA* variant recently predicted with a minigene splicing assay to cause the partial deletion of the adjacent exon and to introduce the p.Gly163Leufs*2 protein defect [20]. All investigated pathophysiologic pathways linking the *GLA* gene variant to the clinical and pathological phenotype concordantly supported the diagnosis of AFD, as elegantly discussed by Oliveira JP et al. [21].

The precise diagnosis and correct classification of gene variants provide the discriminating key for clinically actionable (LP/P) and non-actionable (VUS, LB/B) gene variants [16]. In the last decade, the expansion of population databases, the numerous clinical studies with longitudinal monitoring of families, the increase in knowledge addressing the functional effect of variants in the *GLA* gene, and, in our experience, in vivo tissue studies, either EMB or extracardiac biopsies [6,14], have contributed to the re-classification of numerous gene variants originally interpreted as disease-causing but subsequently recognized as either VUS or LB/B [20]. Clinical actionability of gene variants is essential for treatment decisions. Although the diagnosis of AFD can be obtained with certainty with enzymatic tests in the male patient and in vivo evidence of GB3 accumulation, the genetic test is essential both for family screenings and for therapeutic options, in particular for variant-dependent, chaperone-based therapeutic decisions which in our family cannot be offered because the variant is not amenable to this treatment [22]. Therefore, when the proband of the family is a heterozygous female, the carrier of a novel or ultrarare or ambiguous *GLA* variant, and with non-informative enzyme activity assay, the precise diagnosis is achieved when clinical traits and actionable variants in the *GLA* gene are associated with the evidence of substrate accumulation, concordantly demonstrating the unequivocal interpretation of the variant pathogenicity. Additional in vitro tests exploring the effects of non-canonical splice variants may further contribute to the certainty of clinical actionability [20].

The *GLA* gene databases include hundreds of pathogenic variants associated with classical AFD and late-onset cardiac variants [23]. For numerous validated variants, the genetic test in patients and families with phenotypes consistent with the disease, the measurement of enzyme activity in males, and circulating LysoGB3 may be sufficient for diagnosis and monitoring [4]. However, many variants are unique and described in non-replicated case reports. The demonstration of intracellular accumulations of GB3 remains a key to the pathogenicity of new or ultrarare ambiguous *GLA* variants. In these cases, the addition of the ACMG PS3 criterion to the a priori criteria based on computational algorithms, prediction tools, and databases provides certain diagnoses, resolving the uncertainty of the variant interpretation.

The ClinVar database (2024) currently includes 1041 *GLA* variants, of which: 26 (2.5%) are splice variants that affect canonical splice sites, and 141 are intronic variants (13.5%) potentially affecting splicing; a few of them are deep intronic variants, others affect non-canonical splice sites as for the herein reported variant [20]. Out of the overall intronic variants, 12 are classified as LP/P and 21 as VUS (Table 1). The c.547+3A>G variant is described as VUS but our data prove its pathogenicity.

Overall, when a unique or ultra-rare ambiguous *GLA* variant is identified in the absence of other LP/P variants in the same gene and variant- and gene-level data are insufficient to resolve the uncertainty, a GB3-positive tissue biopsy can meet the PS3 pathogenic criterion, providing an unambiguous diagnosis of AFD.

## Figures and Tables

**Table 1:** P/LP and VUS intronic *GLA* variants in ClinVar database.

Likely Pathogenic/Pathogenic	Variants of Uncertain Significance	
c.1000−10G>A c.19_369+582del c.194+1222_998dup c.195−89_712del c.370−530_1279del c.370−532_1278del c.639+4A>T c.640−801G>A c.640−814T>C c.640−859C>T c.801+3A>G c.802−3_802-2del	c. −1A>C c. −1A>G c. −2C>T c. −79G>A c. −7C>T c.195−13_195-12delinsGC c.195−9C>A c.369+3G>A c.369+4A>T c.369+5G>T c.547+397G>A c.547+3A>G	c.547+4A>C c.548−10T>A c.548−3C>A c.639+3G>A c.639+5G>A c.639+852_639+855del c.640−336T>C c.640−4A>C c.802−14A>T

## Data Availability

A repository of light, electron and immunoelectron microscopy pictures with corresponding positive controls for each biopsy is available under reasonable request to the corresponding author.
